# Deciphering the correlations between aging and constipation by metabolomics and network pharmacology

**DOI:** 10.18632/aging.202340

**Published:** 2021-01-10

**Authors:** Xiaojie Liu, Di Zhao, Sijun Zhao, Zhenyu Li, Yulan Wang, Xuemei Qin

**Affiliations:** 1 Modern Research Center for Traditional Chinese Medicine, Shanxi University, Taiyuan 030006, Shanxi, China; 2 Institute of Biomedicine and Health, Shanxi University, Taiyuan 030006, Shanxi, PR China; 3 Department of Pharmacology, Shanxi Institute for Food and Drug Control, Taiyuan 030001, Shanxi, China; 4 Singapore Phenome Center, Lee Kong Chian School of Medicine, Nanyang Technological University, Singapore 999002, Singapore

**Keywords:** metabolomics, network pharmacology, constipation in the elderly, aging, 1H NMR

## Abstract

From the points of view of phenomena and experience, aging and constipation are inextricably correlated. However, experimental support and underlying mechanisms are still lacking. The purpose of this study is to explore the relationships between aging and constipation from the perspectives of fecal metabolites and network pharmacology. The behavioral analyses of aging and constipation were carried out on both aging rats and constipation rats. We found that aging rats exhibited not only significant aging behaviors but also significant constipation behaviors, while constipation rats exhibited both significant constipation and aging behaviors. Additionally, fecal metabolomics was carried out and found that 23 metabolites were aging-related and 22 metabolites were constipation-related. Among them, there were 16 differential metabolites in common with 11 metabolic pathways. Network pharmacology was applied to construct the target-pathway network of aging and constipation, revealing that pathway in cancer was the most associated signaling pathway. The current findings will provide not only a novel perspective for understanding aging and constipation, but a theoretical association and understanding the traditional Chinese medicine theory and the Western medicine theory about aging and constipation, as well as support for the clinical research and development of medicine related to constipation in the elderly.

## INTRODUCTION

With the worldwide increase in the aging population, more and more attention has been paid to aging [1]. Aging, a nature biological process, is characterized by gradual and progressive declines in physiological functions at multiple levels [2], which results in an increased vulnerability to chronic diseases and even death. While affecting all ages, constipation is a common and serious problem in the elderly that should be paid great attention [3]. The epidemiological data shows that the prevalence of constipation increases with age [4, 5]. Although it is not life-threatening, unlike cardiovascular disease, diabetes, and advanced cancers, constipation greatly influences quality of life in terms of psychological distress and aggravating and/or even causing occurrences of other diseases, e.g. gastrointestinal disorders, colon cancer, etc. [6]. For example, it has been confirmed that constipation positively associates with the increased risk of colon cancer [7–9].

Due to sharing many neurotransmitters and mechanisms of neurotransmission of central nervous system (CNS) and enteric nervous system (ENS), gastrointestinal and CNS pathologies often coexist [10]. For example, patients with chronic constipation generally exhibit obvious psychological distress, including anxiety, depression, obsessive compulsiveness and psychoticism [10–12]. As age increases, brains show characteristic changes of neurodegeneration [13], which can further induce gastrointestinal dysmotility and hormone secretion disorders related [10, 14]. In the theory of traditional Chinese medicine (TCM), more and more viscera functions decline as aging increases, especially spleen and stomach both of which decline prominently. Further, deficiency and dysfunction of spleen and stomach will consequently increase the risk of constipation [15]. Vice versa, constipation can also aggravate agedness process in terms of disturbing the intestinal homeostasis and the normal functions of CNS [16].

Although aging and constipation are easily associated, revealing the scientific relationships and underlying mechanisms remain a challenging task. Both aging and constipation are the external embodiments of the internal microcosmic changes in the body's physiological functions. Therefore, the internal correlations between aging and constipation could be explained from the microscopic metabolites, which will provide a new perspective for revealing the underlying mechanisms. Metabolomics has emerged as a powerful tool for identifying global changes of differential metabolites and metabolic pathways in response to exogenous stimuli, as well as for understanding the complex underlying relationships between aging and constipation [17]. As a new strategy, network pharmacology is developed on the basis of system biology and multi-disciplines, and can systematically and comprehensively analyze the relationships of drugs, targets, metabolic pathways, and diseases [18].

D-galactose (D-gal)-induced aging model, a classic and widely used model [19], was applied in this study. The reason is that the impairment of neurogenesis in the dentate gyrus induced by D-gal is similar to the natural aging process in mice or rats [20]. As well, the constipation model was established under the guidance of TCM theory, which could simulate the clinical symptoms of weakness in elderly patients with constipation. In this study, fecal metabolomics and network pharmacology were, for the first time, used to demonstrate the relationships between aging and constipation, and the underlying mechanisms. Firstly, an aging model and a constipation model were constructed, respectively. And then, behavioral indicators of aging and constipation were measured and analyzed for aging model rats and constipation model rats, providing behavioral correlations at a macro level. An NMR-based metabolomics approach was then applied to find the overlapped and the differential metabolites and metabolic pathways involving in aging and constipation. Additionally, network pharmacology was used to predict the disease targets and metabolic pathways of the two diseases, as well as to construct the corresponding networks. On top of these, the relationships of aging and constipation were systematically analyzed at the different levels, i.e. macro levels of classic behaviors, metabolite levels, metabolic pathways and “disease-target” networks. The results herein will provide not only experimental support for deeply understanding the internal relationships of aging and constipation and the underlying mechanisms, but also theoretical support for associating and understanding the TCM theory and the Western medicine theory about aging and constipation.

## RESULTS

### The effects of aging and constipation on the body-weight gains and food intakes of rats

**Figure 1:**
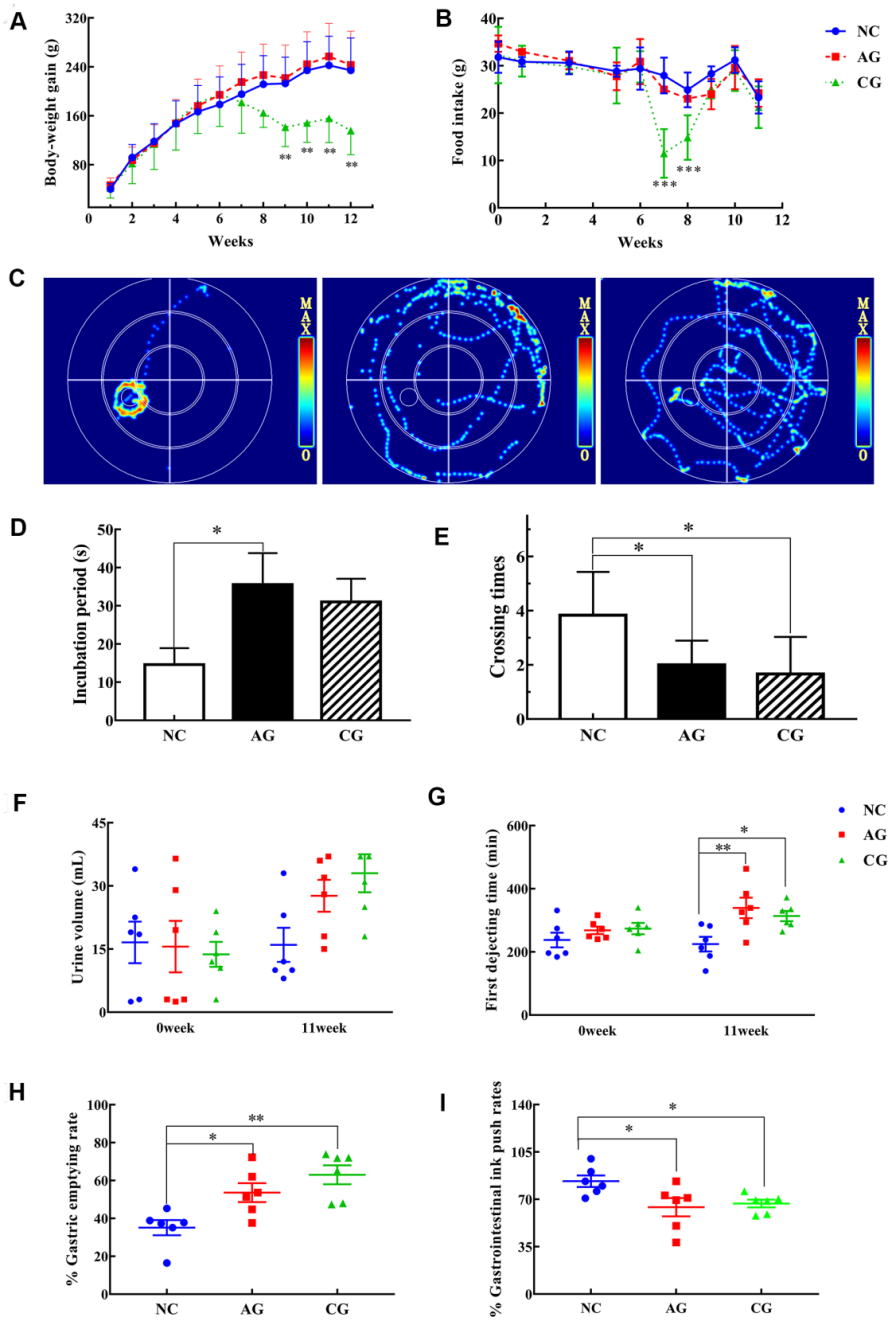
**The results of behavioristics based on the negative control group (NC), the aging model group (AG) and the constipation model group (CG).** (**A**) Body-weight gain; (**B**) Food intake; (**C**) Typical swimming heat map on the spatial probe ability of rats in the fourth day; (**D**) Escape latency; (**E**) The number of crossing the platform; (**F**) Urine volume collected for 12 hours; (**G**) First dejecting time; (**H**) Gastric emptying rates; (**I**) Gastrointestinal ink push rates. Data were presented as the mean ± S.E. (n=6). *P < 0.05, **P < 0.01, compared with NC.

The initial body weights did not differ among the three groups (Figure 1A). The body-weight gains of rats in the NC and the AG groups increased generally as the time passed, while no significant differences were observed. However, the body weight gains of rats in the CG group were significantly lower than that of the NC group. Meanwhile, no significant difference in the food intake was observed among the three groups (Figure 1B). Noticeably, after one-week administration of white vinegar, rats in the CG group experienced sharp decrease in food intakes, which eventually returned to the same levels as the control rats.

These results showed that the 11-week treatment of D-gal did not significantly influence the body weight and food consumption of rats. By contrast, white vinegar had a provoking effect on rats with decreasing their food intakes in a short period.

### Aging and constipation decreased the locomotor activity and the spatial learning and memory ability of rats

OFT was conducted to detect the locomotor activity and exploration behaviors of rats. As compared to the control rats, the total distances and the rearing numbers of rats in both the AG and the CG groups significantly decreased (Table 1).

**Table 1:** Behavioral parameters of the control rats, the aging rats and the constipation rats in the open-field test (n=6, mean ± S.E.).

**Time (weeks)**	**Group**	**Duration time in the center (s)**	**Grooming time (s)**	**Total distance**	**Rearing**
0	NC	1.26 ± 0.22	15.03 ± 13.12	54.43 ± 20.49	8.00 ± 3.69
	AG	1.33 ± 0.41	12.77 ± 5.09	55.88 ± 20.57	10.33 ± 4.68
	CG	1.38 ± 0.42	13.00 ± 13.81	55.38 ± 26.33	10.00 ± 7.92
11	NC	1.12 ± 0.47	18.66 ± 3.42	55.33 ± 13.41	11.00 ± 3.69
	AG	0.80 ± 0.39	11.90 ± 10.07	32.33 ± 26.38*	6.33 ± 4.59*
	CG	1.14 ± 0.52	22.94 ± 14.47	12.17 ± 16.52**	2.33 ± 3.39***

* *P* < 0.05, ** *P* < 0.01 as compared with NC.

Abbreviations: NC, the negative control group; AG, the aging model group; CG, the constipation model group.

MWM tests were performed to assess the spatial cognitive and learning impairment of rats. Firstly, the movement trajectories of the rats were observed. Regarding the swim paths and the heat-map summary of rats on the 4^th^ day, rats in the AG and the CG groups presented a more scattered route and took longer time to find the targets than rats in the NC group did (Figure 1C). Secondly, concerning the space exploration, the time required for the aging and the constipation rats to cross the previous location of the platform significantly increased (Figure 1D), while they spent less time to cross the platform location than control rats (Figure 1E), indicating a decrease of spatial memory of rats that induced by aging and constipation.

### Aging and constipation induced gastrointestinal dysfunctions of rats

The gastrointestinal transit trial and the gastric and small intestinal movement trial were further performed to detect the defecation status of rats. Before the experiments, neither the initial urine volume, nor the final urine volume differed among the three groups (Figure 1F). There was no significant difference in the initial defecating time of the first black feces among the three groups (Figure 1G).

After a 11-week treatment, the defecation time of rats in the AG and the CG groups was significantly prolonger than that of control rats. The gastric emptying rates of rats of the two treatment groups were also significantly longer than those of the NC group (P < 0.05; P < 0.01) (Figure 1H). In addition, the gastrointestinal ink push rates of the AG and the CG groups significantly decreased (Figure 1I). These results showed that both aging and constipation could significantly weaken the gastrointestinal motility of rats.

### Aging and constipation induced oxidative damage of rats

To verify the level of oxidative stress injury in rats, serum samples of rats were collected for biochemical assays. As compared to the control rats, the levels of SOD, CAT and GSH-Px significantly decreased in the aging rats and the constipation rats, while the activity of MDA increased (Table 2). Overall, both the aging rats and the constipation rats showed significant oxidative damage, which could not effectively active antioxidant factor and reduce the accumulation of reactive oxygen species (ROS).

**Table 2:** Biochemical parameters in the serum samples of the control and the aging groups (n=6, mean ± S.E.).

**Group**	**SOD (U/mL)**	**MDA (nmol/mL)**	**CAT (U/mL)**	**GSH-Px (U/mL)**
NC	619.78 ± 19.49	5.35 ± 0.47	14.69 ± 1.86	1896.08 ± 90.90
AG	533.96 ± 16.29^**^	7.38 ± 0.66^*^	5.50 ± 0.75^**^	1572.55 ± 61.11^*^
CG	371.10 ± 2.71^***^	7.31 ± 0.55^*^	1.92 ± 1.71^***^	1545.10 ± 75.98^*^

*P < 0.05, **P < 0.01 as compared with NC.

Abbreviations: NC, the control group; AG, the aging group; CG, the constipation group.

### Aging and constipation significantly decreased the express of caspase-3 in the hippocampal tissues of rats

**Figure 2:**
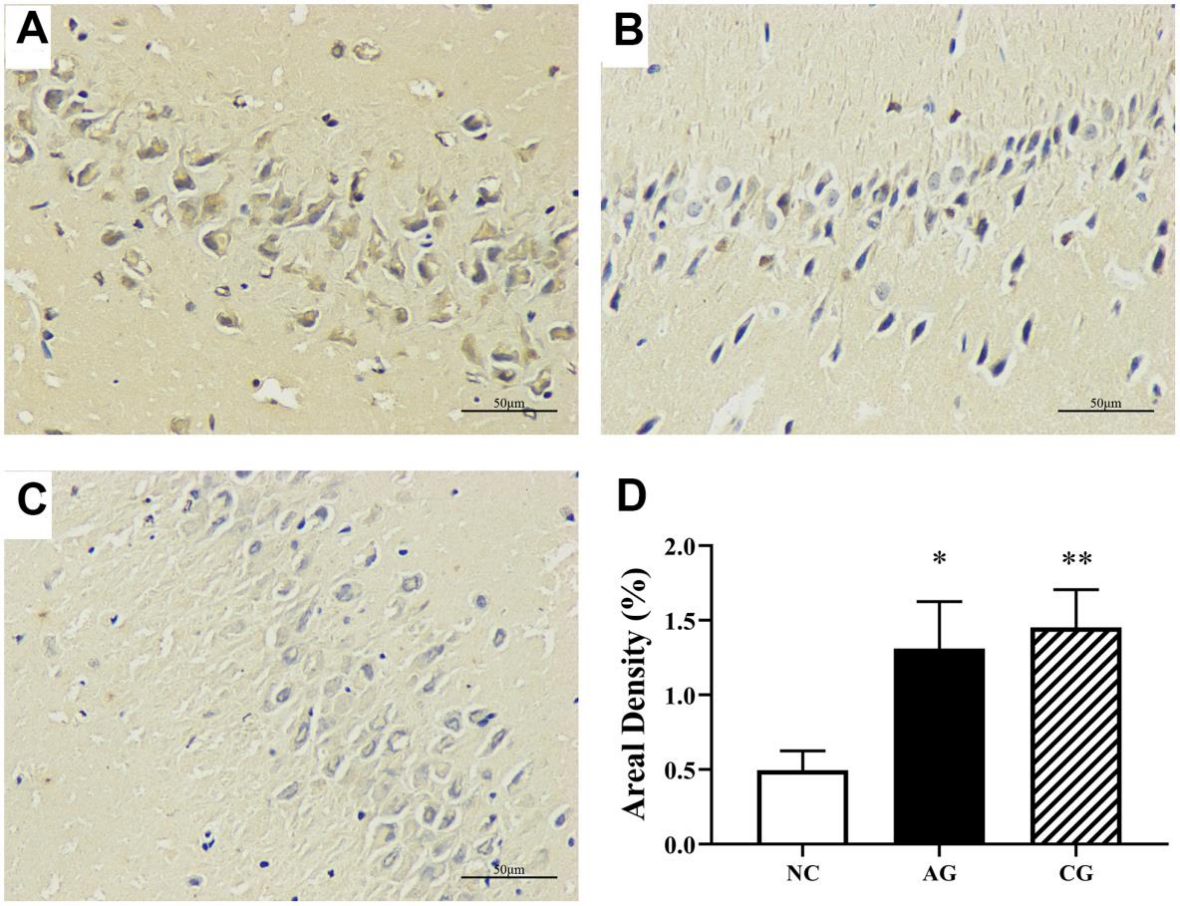
****Hippocampal immunohistochemical analysis of the negative control group (**A**), the aging group (**B**) and the constipation group (**C**) under an optical microscopy (400 × magnification) and their semi-quantification (**D**). Data were presented as the mean ± S.E. (n=3).

Immunohistochemical staining of hippocampal tissues of rats was examined to further investigate the expression of caspase-3, a target protein. This is especially useful for assessing the progression of diseases and treatment of the effects of diseases. Immunohistochemically, high expressions of caspase-3 (Figure 2D) were found in hippocampal tissues of the aging rats and the constipation rats than those of the control rats. As demonstrated in Figures 2A–2C, there was no obvious abnormality or tissue damage occurred in the hippocampal tissues of the control group, while pathological changes occurred in the model rats.

### Aging and constipation significantly perturbed the metabolite profiles of rats

**Figure 3:**
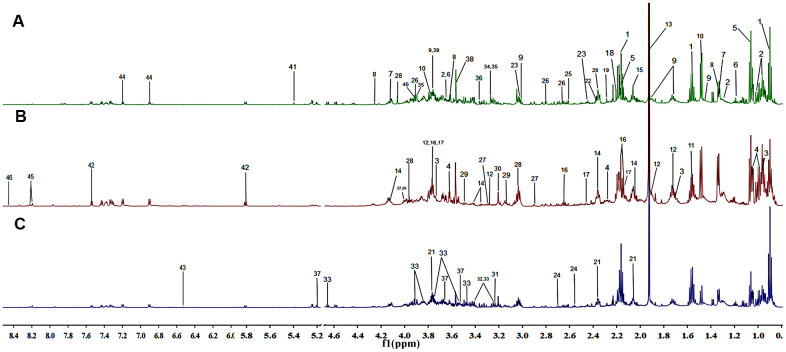
Representation of the 600 MHz ^1^H NMR spectra of the fecal samples collected from the negative control group (**A**), the aging group (**B**) and the constipation group (**C**). The assignments of 46 metabolites are given in Table 3.

Representative ^1^H NMR CPMG spectra of fecal extracts collected from the NC, the AG and the CG groups were shown with major metabolites being labeled (Figure 3). In total, 46 metabolites were identified (Table 3).

**Table 3:** ^1^H NMR assignments of major metabolites from fecal samples of rats.

**NO.**	**Metabolites**	**δ ^1^H (multiplicity ^a^)**	**NO.**	**Metabolites**	**δ^1^H (multiplicity ^a^)**
1	Butyrate	0.90(t), 2.16(t), 1.56(m)	24	Citrate	2.54(d), 2.67(d)
2	Isoleucine	0.96(t), 1.01(d), 1.27(m), 3.65(d)	25	Methylamine	2.61(s)
3	Leucine	0.97(t), 1.69(m), 3.72(t)	26	Aspartate	2.66(dd), 2.80(dd), 3.91(dd)
4	Valine	0.99(d), 1.04(d), 2.27(m), 3.62(d)	27	Asparagine	2.87(m), 3.03(m), 4.00(m)
5	Propionate	1.06(t), 2.17(q)	28	Creatinine	3.04(s), 3.96(s), 4.05(s)
6	Ethanol	1.18(t), 3.66(q)	29	Phenylalanine	3.17(dd), 3.33(m), 3.51(s), 3.99(dd), 7.33(t), 7.38(m), 7.43(t)
7	Lactate	1.32(d), 4.13(q)	30	Choline	3.21(s)
8	Threonine	1.34(d), 3.59(d), 4.26(m)	31	Carnitine	3.23(s)
9	Lysine	1.47(m), 1.72(m), 1.89(m), 3.01(t), 3.76(t)	32	Taurine	3.25(t), 3.41(t)
10	Alanine	1.48(d), 3.81(q)	33	Glucose	3.25(t), 3.42(dd), 3.47(dd), 3.54(dd), 3.75(m), 3.84(m), 3.91(dd), 4.65(d), 5.24(d)
11	Citrullinine	1.56(m)	34	TMAO ^c^	3.26(s)
12	Arginine	1.72(m), 1.90(m), 3.25(m), 3.76(m)	35	Betaine	3.26(s), 3.90(s)
13	Acetate	1.92(s)	36	Scyllo-Inositol	3.37(s)
14	Proline	2.05(m), 2.36(m), 3.35(t), 3.42(t), 4.12(m)	37	*α*-xylose	3.55(dd), 3.67(m), 5.21(t)
15	N-acetyl-GT ^b^	2.06(s)	38	Glycine	3.55(s)
16	Methionine	2.14(s), 2.17(m), 2.64(t), 3.77(m)	39	Mannitol	3.77(dd)
17	Glutamine	2.15(m), 2.46(m), 3.77(m)	40	Glycolate	3.93(s)
18	Acetone	2.21(s)	41	Allantoin	5.39(s)
19	Acetoacetate	2.30(s)	42	Uracil	5.81(d), 7.54(d)
20	Pyruvate	2.35(s)	43	Fumarate	6.53(s)
21	Glutamate	2.36(m), 2.06(m), 3.78(m)	44	Tyrosine	6.90(d), 7.2(d)
22	Succinate	2.39(s)	45	Hypoxanthine	8.2(s), 8.22(s)
23	2-oxoglutarate	2.45(t), 3.02(t)	46	Formate	8.46(s)
^a^: s, singlet; t, triple; q, quartet; m, multiple ^b^: N-acetyl-glycoprotein ^c^: Trimethylamine-N-oxide.

**Figure 4:**
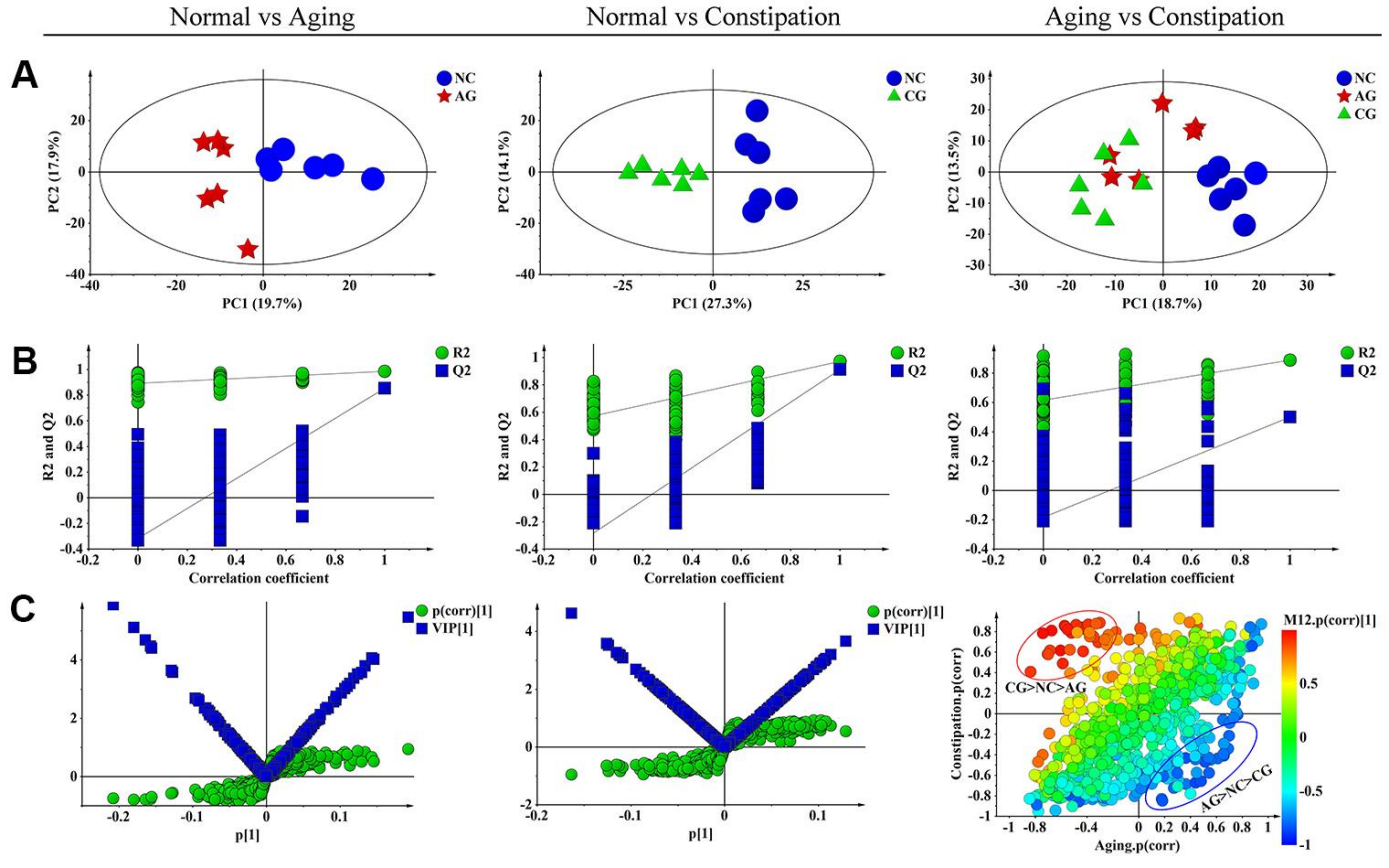
**Multivariate data analysis of ^1^H NMR fecal extract spectra among the negative control group (NC), the aging model group (AG) and the constipation model group (CG).** (**A**) PCA scores plot of fecal specimens based on NC (blue dots), AG (red boxes) and CG (green triangles). (**B**) Statistical validation of the corresponding PLS-DA model by permutation analysis (permutation No.: 200). R^2^ is the explained variance, and Q^2^ is the predictive ability of the model. (**C**) The corresponding S-plot and VIP value of fecal specimens in aging and constipation, as well as SUS-plot plots showing the discriminations between aging and constipation. PCA, principal component analysis; PLS-DA, partial least squares discriminant analysis; SUS-plot, shared-and-unique-structures plot.

In the PCA score plot, we observed obvious separations between the NC group and the AG group, as well as between the NC group and the CG group (Figure 4A). Yet, the AG group and the CG group did not obviously separate with each other (Figure 4A). Meanwhile, PLS-DA was performed to maximize the differences of the metabolic profiles and showed the intergroup metabolic differences. These PLS-DA models from NC vs. AG (R^2^Y = 0.986, Q^2^ = 0.855) and NC vs. CG (R^2^Y = 0.967, Q^2^ = 0.85) were valid without over-fitting (Figure 4B).

### Identification of differential metabolites associated with aging and constipation

S-plot analysis was performed firstly (Figure 4C), where both VIP > 1.2 and *P* < 0.05 were used to screen differential metabolites associated with aging or constipation. The levels of 23 fecal metabolites were found to be significantly changed in the AG group as compared to the NC group, including higher levels of acetate, α-xylose, choline, glucose, lactate, proline and threonine, but lower amounts of 2-oxoglutarate, acetone, alanine, asparagine, aspartate, citrate, creatinine, glutamate, isoleucine, leucine, lysine, methionine, phenylalanine, pyruvate and scyllo-inositol (Table 4). In the corresponding S-plot of the CG group and the NC group, 22 metabolites were identified to be responsible for distinguishing the two groups (Figure 4C), including elevated levels of acetate, α-xylose, arginine, butyrate, carnitine, choline, ethanol, glucose, phenylalanine, taurine and trimethylamine-N-oxide (TMAO), as well as decreased levels of alanine, asparagine, aspartate, citrate, creatinine, glutamate, leucine, methionine, proline, pyruvate and isoleucine.

**Table 4:** Changes of differential metabolites in the aging and the constipation groups.

**No.**	**Fecal metabolites**	**Aging**	**Constipation**
1	2-oxoglutarate	↓	-
2	Acetate	↑	↑
3	Acetoacetate	↑	-
4	Acetone	↓	-
5	Alanine	↓	↓
6	*α*-xylose	↑	↑
7	Arginine	-	↑
8	Asparagine	↓	↓
9	Aspartate	↓	↓
10	Butyrate	-	↑
11	Carnitine	-	↑
12	Choline	↑	↑
13	Citrate	↓	↓
14	Creatinine	↓	↓
15	Ethanol	-	↑
16	Glucose	↓	↑
17	Glutamate	↓	↓
18	Isoleucine	↓	↓
19	Lactate	↑	
20	Leucine	↓	↓
21	Lysine	↓	-
22	Methionine	↓	↓
23	Phenylalanine	↓	↑
24	Proline	↑	↓
25	Pyruvate	↓	↓
26	Scyllo-Inositol	↓	-
27	Taurine	-	↑
28	Threonine	↑	-
29	Trimethylamine-N-oxide	-	↑

“-”: Not changed;

as compared to control group, “↑”: increased; “↓”: decreased.

16 differential metabolites were over-lapped involved in both aging and constipation, including acetate, α-xylose, alanine, asparagine, aspartate, citrate, creatinine, choline, glutamate, glucose, isoleucine, leucine, methionine, proline, phenylalanine and pyruvate (Table 4).

### Unique and sharing differential metabolites associated with aging and constipation

A SUS-plot was then performed to integrate the two OPLS models (Figure 4C). In the SUS-plot, the variables that related to the influence of aging were located in lower right quadrants while constipation-related variables were located in upper left quadrants. According to the SUS-plot analysis, three unique metabolites of aging include lactate, acetoacetate and formate, while there are 6 unique metabolites associated with constipation including arginine, carnitine, proline, scyllo-inositol, valine, and α-xylose. The 18 shared metabolites of aging and constipation included alanine, allantoin, asparagine, aspartate, butyrate, choline, citrate, citrullinine, glucose, glutamine, glycine, hypoxanthine, mannitol, methionine, pyruvate, phenylalanine, threonine and uracil.

### Unique and sharing metabolic pathways perturbed by aging and constipation

**Figure 5:**
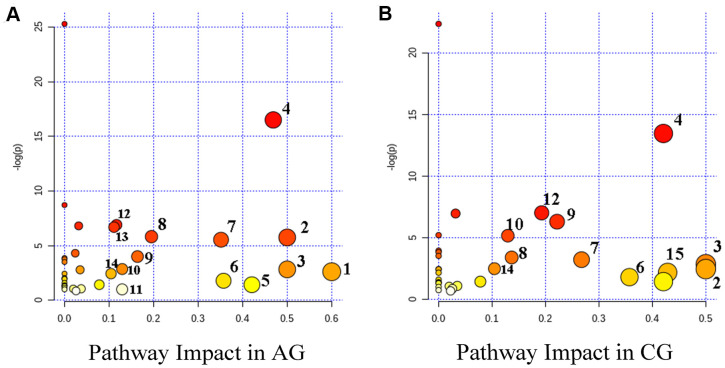
****Summary diagram of the pathway analysis of the differential metabolites with MetPA in the aging group (AG) (**A**) and the constipation group (CG) (**B**). The size and the color of each circle were based on the pathway impact value and *P*-value, respectively. Metabolic pathways are as follows: 1, Synthesis and degradation of ketone bodies; 2, D-glutamine and D-glutamate metabolism; 3, Phenylalanine, tyrosine and tryptophan biosynthesis; 4, Alanine, aspartate and glutamate metabolism; 5, Starch and sucrose metabolism; 6, Phenylalanine metabolism; 7, Pyruvate metabolism; 8, Citrate cycle (TCA cycle); 9, Arginine and proline metabolism; 10, Glycolysis / Gluconeogenesis; 11, Inositol phosphate metabolism; 12, Arginine biosynthesis; 13, Butanoate metabolism; 14, Cysteine and methionine metabolism; 15, Taurine and hypotaurine metabolism.

Further analysis revealed that 14 and 12 metabolic pathways were involved in aging and constipation, respectively. There were 11 overlapping metabolic pathways including D-glutamine and D-glutamate metabolism, phenylalanine, tyrosine and tryptophan biosynthesis, starch and sucrose metabolism, phenylalanine metabolism, arginine and proline metabolism, cysteine and methionine metabolism, arginine biosynthesis and pyruvate metabolism (Figure 5).

### Correlations between aging and constipation from the perspective of behaviors

**Figure 6:**
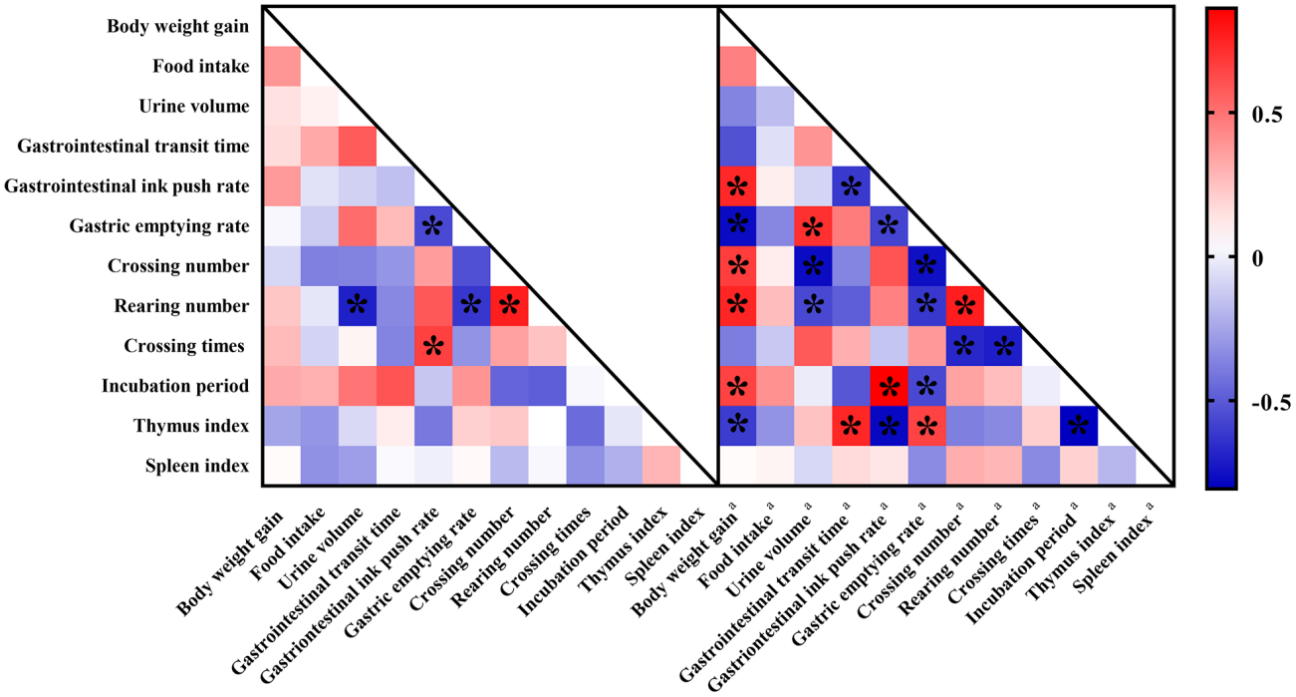
**Correlation heat map color-coded by the strengths of Pearson correlation coefficients (r) between behaviors.** Cut-off values of |r| > 0.5 and *P* < 0.05 have been used. “*” means significantly differences. Red boxes indicate positive associations while blue boxes indicate negative associations. Parameters labeled with “a” indicated fecal biomarkers involving in constipation while metabolites without any labels indicated fecal biomarkers involving in aging.

There are often internal links between animal behaviors (Figure 6). In this study, we found that in the AG group, the number rearing times was negatively correlated with urine volume and gastric emptying. There was a significant negative correlation between intestinal propulsion rate and gastrointestinal emptying rate in aging rats. In addition, the number of crossing platforms in the MWM test was negatively correlated with the small intestine propulsion rate, as well as with the standing number in the OFT.

For behaviors of constipation rats, more correlations were found. Firstly, body-weight gains of constipation rats were significantly associated with six behavioral parameters which could be assigned to autonomic mobility, excretion behaviors, learning and memory ability, and thymus index. There was a significant positive correlation between urine volume and gastric emptying. Escape incubation period was significantly correlated with gastric emptying and intestinal propulsion. In addition, there was also a significant correlation between autonomic mobility and thymus index.

### The GO enrichment analysis and the pathway enrichment analysis of aging and constipation

**Figure 7:**
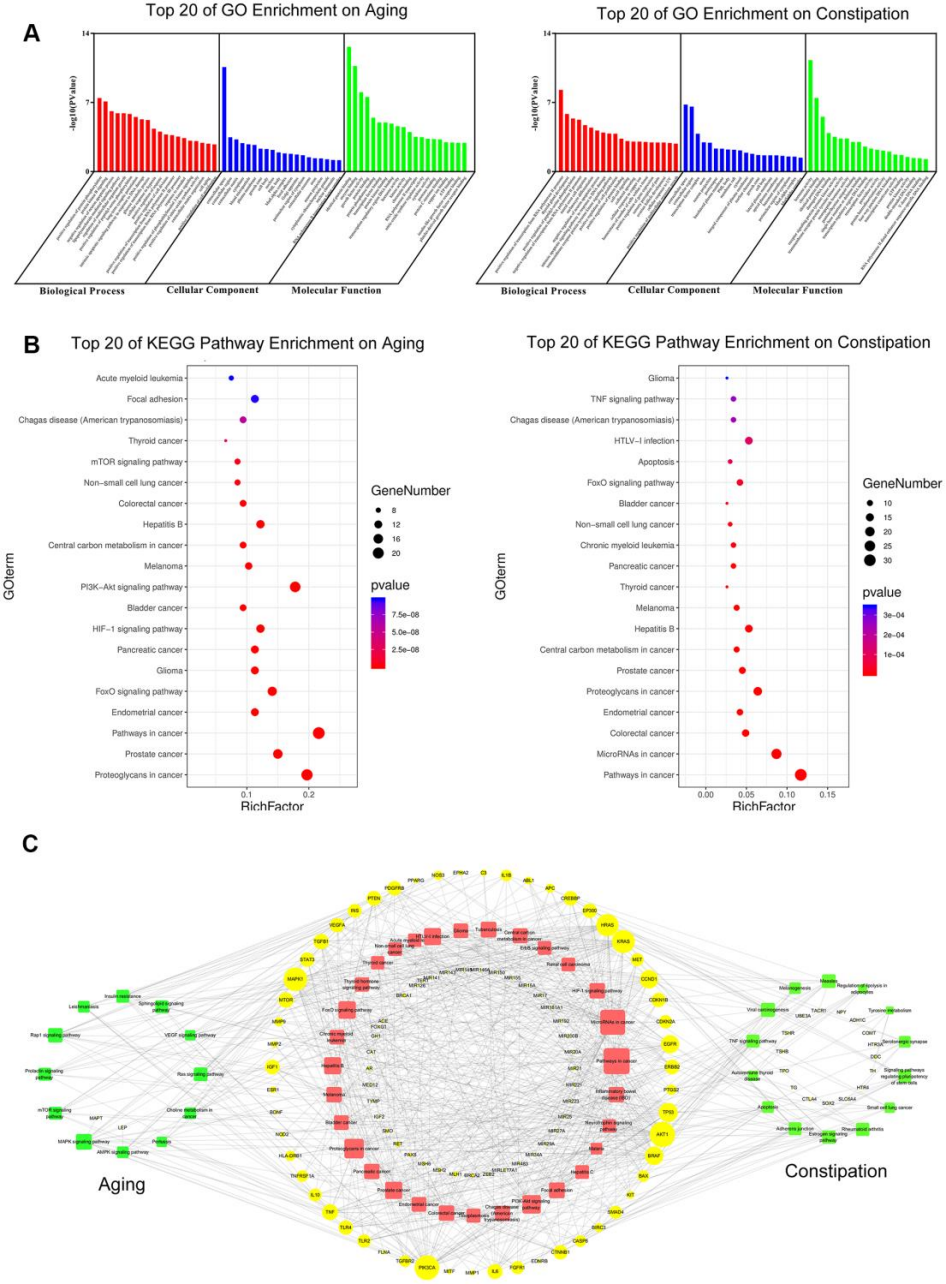
GO enrichment analysis (**A**) and KEGG pathway analysis (**B**) of potential targets of aging and constipation. The size of the bubbles in each bubble chart represents the gene counts of this entry. The colors from cold to warm represent the *P* values from large to small. Each bubble chart is sorted by *P* value. GO: gene ontology; KEGG: kyoto encyclopedia of genes and genomes. (**C**) The target-pathway network diagram of aging and constipation. The yellow labels and the blue labels represent disease targets and signing pathways, respectively. The red labels and the green labels represent the common signaling pathways between aging and constipation, and the unique disease targets for aging or constipation, respectively. The size of each label represents its degree. The thickness and color of the lines represent edge. The edges represent the interactions between them and node sizes are proportional to their degree. The blue nodes and the red nodes represent targets pathways, respectively.

Firstly, through the analysis of disease targets, we found 23,989 aging-related targets and 4636 constipation-related targets. Afterwards, the GO enrichment analysis and the pathway enrichment analysis of screened targets were conducted via a functional annotation tool of DAVID Bioinformatics Resources 6.8. to identify the biological characteristics of putative targets of aging and constipation in details. In total, 154 biological process terms (BP), 23 cellular component terms (CC), and 25 molecular function terms (MF) were identified in aging. As for constipation, there were 132 BP terms, 32 CC terms, and 26 MF terms. According to the requirements of screening criteria, i.e. Count 2 and EASE scores 0.1, the top 20 significantly enriched terms in BP, CC, and MF categories were shown in Figure 7A.

To explore the underlying involved pathways of aging and constipation, KEGG pathway analysis of involved targets was conducted. 32 pathways were involved in both aging and constipation. The top 20 significantly enriched pathways of aging and constipation were shown in Figure 7B.

### Correlations between aging and constipation from the disease-target network perspective

To further characterize the correlations between aging and constipation in molecular mechanisms, a target-pathway network was performed on the basis of the involved proteins and their corresponding significant signaling pathways (Figure 7C). This network consists of 171 nodes, of which 113 are protein nodes and 58 are pathway nodes. Among these potential pathways, pathway in cancer was the most significant one with the highest degree value. Among these potential targets, AKT1, MAPK1, PIK3CA, TP53, BRAF, mTOR, TNF, and CCND1 were identified as relatively high-degree targets, which promoted the development of aging and constipation.

## DISCUSSION

Aging and constipation are easily and closely linked. Yet, there is still a lack of theoretical evidence to support such a correlation. In this study, firstly, we successfully established two independent models, namely the aging model and the constipation model. And then, we further evaluated the learning cognitive ability, the gastrointestinal motility and the locomotor activity of rats, from a macro level. Furthermore, of note, from a microcosmic view, an NMR based metabolomics approach coupled with multivariate data analysis was applied to analyze the common points between aging and constipation in the metabolites and metabolic pathways. In addition, considering the importance of the consistency of the pathogenesis between diseases in finding the commonality, network pharmacology was also applied to find the disease targets and metabolic pathways of the two diseases, aiming at further clarifying the connections between aging and constipation. The results show that there are many common grounds between aging and constipation, including but not limiting, behaviors, oxidative stress, expression of caspase-3 proteins, metabolites, disease targets and metabolic pathways. The results of this study prove and confirm the intrinsic correlations between aging and constipation, and provide evidence for their mutual clinic symptoms, clinical treatments and drug discovery.

### Correlations of behavioral abnormalities across aging and constipation rats

Normally, behaviors represent the final output of the brain work [21, 22]. Therefore, we tested the learning and memory abilities, the excretion abilities and the autonomic activities of aging rats and constipation rats to visualize their underlying relationships, as well as to reveal the corresponding mechanisms.

Learning and memory ability is a powerful indicator of cognitive functions in the CNS [23, 24]. MWM has been widely used to measure spatial navigation and memory in rats [25]. In this study, both the aging and the constipation rats showed worse spatial and learning memories than the control rats (Figure 1), indicating that CNS functions were damaged by aging and constipation.

Parameters, including gastric emptying rates, gastrointestinal ink push rates and gastrointestinal transit time, have been widely used to detect the effects of laxative agents on constipation [26]. Herein, these parameters were simultaneously measured to investigate the impacts of aging and constipation on gastric motility of rats. Aging is accompanied by a large number of organ weaknesses, especially the function declines in gastrointestinal tract and kidney [5, 27]. The increase of urine volume is a common phenomenon in the elderly, which may be an early symptom of kidney diseases. Gastrointestinal dysfunction and lack of defecation power are the main causes of constipation in the elderly [28]. In this study, both aging and constipation rats showed decreases in gastrointestinal motility and defecation ability, as suggested by the results that gastrointestinal transit time was significantly prolonged, gastrointestinal emptying rate was increased and intestinal propulsion rate was significantly reduced. Gastric peristalsis and contractility were also weakened in the elderly [29]. Gastric myoelectrical disturbances may lead to gastric motor abnormalities and disturb gastric emptying. The underlying mechanisms of gastric motility reducing and gastric emptying reducing of aging may be explained by an abnormality of gastric electro-activity [30].

OFT was used to test the autonomous motor abilities of rats. Both aging rats and constipation rats had significantly higher numbers of crossing times and longer total distances than the control rats (Table 1), suggesting a significant reduce of activity ability. As skeletal muscle mass and muscle strength decrease with age [31], the autonomic activities of aging rats also decline. The physical weaknesses of bodies positively associate with gastrointestinal diseases, e.g. gallbladder disease, colon cancer, and constipation [32]. In this study, a decrease in locomotor activity in aging rats and constipation rats indicates remarkable declines of gastrointestinal functions.

Pearson’s correlation analysis showed that, for aging rats, the aging index reflecting cognitive ability significantly positively correlated with the constipation index reflecting gastrointestinal motility, indicating that constipation phenomena become worse as age increases. There was a significant positive correlation between the number of times of standing and the number of crossing platforms, which indicated that with age, the ability of autonomous movement also decreased. Similarly, an inverse relationship between standing times and the gastric emptying rate indicates that the occurrence of constipation is accompanied by the decline of the ability of autonomous activity.

The behavioral relationships occurred in the constipation rats were the same as that in the aging rats. Both aging rats and constipation rats showed a common bias and relationship in the three behavioral tests, strongly demonstrating mutual influences of aging and constipation on each other.

### Correlations of oxidative damage across aging and constipation rats

Oxidative stress has been proved to be a promoter of many diseases. Neurodegenerative diseases, e.g. aging, are caused due to excess oxidative stress and alter the functions of CNS. In addition, the occurrence and the development of constipation are also closely related to oxidative stress. Increased oxidative stress has been testified to disrupt mucosal barrier function of intestinal epithelial cells and increase intestinal permeability [32]. CAT is found to involve in the reduction of lipids and hydrogen peroxides [33]. SOD and GSH-Px are well-known radical superoxide scavengers against oxidative stress through reducing ROS. MDA, the end decomposition product of lipid hydroperoxides, is an indirect indicator of oxidative damage in organism [34]. In the present study, the serum levels of SOD, CAT and GSH-Px of both the aging rats and the constipation rats significantly decreased as compared to control rats, while the level of MDA increased. The significant changes of these oxidative parameters suggested an occurrence of serious oxidation in the aging rats and the constipation rats.

### Apoptosis caused by aging and constipation

Apoptosis plays an important role in many physiological and pathological processes such as growth, development and aging. Caspase-3 is the most important terminal shear enzyme during apoptosis, which is expressed in apoptotic cells, apoptotic bodies, etc. [35]. The CA3 area of the hippocampus is regarded to be tightly related to learning and memory ability. In this study, both AG and CG rats showed significantly higher expression of caspase-3 in the CA3 region than the control rats, indicating that cell apoptosis significantly occurred in the hippocampus tissues of aging rats and constipation rats. There are many factors that trigger apoptosis, among which oxidative stress is an important link. Aging is associated with an increased level of ROS in mitochondria. An increase of aging activates apoptosis [36]. In combination with the results of oxidative stress (Table 2), we could conclude that the death of brain cells occurring in the aging and the constipation rats could be resulted from the imbalance of free radicals and antioxidant defenses. Overall, apoptosis has been proved to be linked to oxidative stress and imbalance between generation of free radicals and antioxidant defenses. The results herein further suggest that apoptosis also occurs and involves in both aging and constipation, which could be one of the important mechanisms of aging and constipation.

### Metabolic correlations across aging and constipation rats

### Metabolic changes associated with aging

Development and progression of aging are associated with cellular metabolic changes, which may provide novel insights into pathogenesis. Since food intakes did not differ between the control rats and the aging rats, changes in the fecal levels of amino acids, food breakdown products and metabolites, do reflect changes in the characteristics of the intestinal microbiota associated with age [37].

The depletion of glucose, the increase of lactate, as well as the negative correlations of the two metabolites (Figure 4A), indicate an increased dependency of aging on glycolysis. This is also supported by the elevated levels of 2-oxoglutarate, pyruvate and citrate, indicative of a decrease activity of TCA cycle necessary to maintain physiological activities of the body. In addition, glutamate and alanine were also significantly decreased, which positively correlated with the level of glucose in aging. Glutamate is regarded as another main pillar for energy production in proliferating cells. Subsequent transamination between glutamate and pyruvate catalyzed by alanine transaminase produces alanine and α-ketoglutarate. The latter enters the TCA cycle under an aerobic condition. Therefore, reduced levels of glutamate and alanine suggested a decrease of energy supply in the development of aging.

Higher amounts of amino acids such as leucine and isoleucine were present in the feces of aging rats as compared to the control rats, possibly resulting from malabsorption due to epithelial inflammation and injury caused by aging. Higher levels of aspartate, methionine, threonine, and phenylalanine are associated with dementia [38, 39], possibly because of the effect of aging on neurotransmitter balance in rat brains [40].

A depleted level of creatine in the aging rats has been related to altered energetic transfer processes and may reflect increased activity of creatine kinase. With participating in membrane biosynthesis, choline has been found to be over expressed in aging and highly activated in cell lines. Therefore, an increase of choline level observed in feces could reflect an increase uptake of choline and metabolism of aging cells.

### Metabolic changes associated with constipation

The fecal metabolic profiling from the constipation rats provided abundant data on the status of the gastrointestinal tract metabolites, which reflect environmental factors, genetic and cellular physiological processes [41]. The constipation-related metabolic pathways mainly include energy metabolism, amino acid metabolism, choline metabolism and short chain fatty acid metabolism.

Citrate, an intermediate of TCA cycle, showed a lower level in the feces of constipation rats compared with control rats, suggesting constipation perturbed the energy metabolism. In addition, as the main energy source, the decrease content of glucose further supports the disorder of energy metabolism in constipation. Taurine achieves defecation by increasing gastrointestinal motility in constipation rats [42]. A reduced level of fecal taurine in constipation rats indicated that the gastrointestinal motility of rats was insufficient due to the occurrence of constipation.

Fecal contents of choline are mainly related to dietary intakes, absorption in the small intestine and conversion by gut bacteria to methylamines [43]. Methylamine gas production negatively correlated with colonic transport [44]. Therefore, it is speculated that the occurrence of constipation may decrease the choline level.

SCFAs including acetate and butyrate were found to be significantly disordered in constipation rats. These SCFAs are normally produced in the colon by the gut bacteria *via* complex fermentation of carbohydrate [45]. Especially, butyrate is readily absorbed by the intestinal epithelium and then is utilized as an important energy source for epithelium barrier protection. The depletion of butyrate in constipation rats is likely due to its over-utilization, suggesting a perturbation of SCFA metabolism or a disruption of intestinal micro-ecology environments.

### Correlations of metabolic variations between aging and constipation

In order to find the underlying correlations between aging and constipation from a microscopic perspective, we summarized the changes of fecal metabolites and metabolic pathways. There are 16 overlapping metabolites that involved in both aging and constipation. Except for phenylalanine and proline, the other superposed metabolites showed the same trends. As for metabolic pathways, there are 9 overlapping metabolic pathways of aging and constipation, which are mainly involved in energy metabolism and amino acid metabolism. In addition, a correlation analysis of overlapping metabolites and behaviors show that these changes are consistent.

The results herein suggested that not only the gastrointestinal tract was disordered and the gastric motility was weakened in the process of aging, but also the constipation definitely decreased the learning and memory ability and the autonomous activities. Overall, we could conclude that aging and constipation accelerate or promote the process of each other. Worse, such an acceleration and/or a promotion could bring serious complications, such as cerebral thrombosis, cancer, and so on, thus causing more and more serious damage to bodies.

### Disease targets and signaling pathways involving in aging and constipation

GO enrichment analysis showed that both aging and constipation may occur via enzyme binding, protein binding, and transcription factor binding in cytosol, plasma membrane, and extracellular space [46]. To explore the roles of disease targets in aging and constipation, KEGG pathway analysis was then conducted. The first 20 significantly rich pathways involving in aging and constipation are shown in Figure 6.

For both aging and constipation, pathway in cancer was the most associated signaling pathway. On the one hand, aging is closely related to cancers, both of which have the same physiological mechanisms. For example, the occurrences of various cancers are higher in the elderly than that in other ages [47, 48]. Concerning the treatments, aging related inflammatory cytokines can be used to treat cancers and aging related diseases through targeting at specific inflammatory mediators [49]. On the other hand, constipation is one of the early symptoms of many cancers or an accompanying symptom of cancers [50]. Therefore, it is undoubted that pathway in cancer was the most associated targets for both aging and constipation.

Regarding the pathways in common, 32 target-metabolic pathways were found to be overlapped involving in both aging and constipation (Figure 6). MicroRNAs in cancer, as the common KEGG pathways between aging and constipation, has been found to be involved in many diseases and disorders [51].

Metabolomics based on NMR coupled with MVD analysis has been successfully applied in identifying metabolites that specific metabolic characteristics produced by diseases that interfere with biological pathways. However, some limitations should be noted about this work. With respect to the methodology, it may require other analytical methods such as LC-MS, GC-MS to verify the results. Besides, since brain is the most reflective tissue of degenerative changes, it is necessary to strengthen the analysis of brain in the future work. In addition, considering the fact that the physiological links between aging and constipation are multi-factorial, in the future work, transcriptome and proteomics technologies, with high sensitivity and strong characterization ability, will be applied to fundamentally reveal the correlations between aging and constipation at the levels of genes and proteins.

In summary, in this study, for the first time, taking aging and constipation as the examples, underlying correlations and corresponding mechanisms of the two well-known diseases were demonstrated by two approaches of system biology, i.e. fecal metabolomics and network pharmacology. From both macro level of classic behaviors and micro level revealing by immunohistochemical staining, oxidative stress, metabolic profiles, fecal metabolites, metabolic pathways and disease-target networks and gene function analyses, etc., the common points in pathogenesis of aging and constipation were found. The present findings will not only improve our understanding in the pathogenesis of aging and constipation, as well as in the TCM theory and modern Western medicine theory, but also will provide solid experimental evidence for the development of treatments and clinical practices in the elderly with constipation.

## MATERIALS AND METHODS

### Materials and reagents

D-galactose (D-gal, No. WXBC6583V) was purchased from the Sigma Chemical Co. Ltd. (St Louis, USA) and dissolved in 0.9% saline at a concentration of 300 mg/mL. White vinegar was purchased from the Shanxi Mature Vinegar Group Co. Ltd. (Taiyuan, China). Powder Activated Carbon (PAC) was purchased from the Fuchen Chemical Reagent Co. Ltd. (Tianjin, China). Deuterium oxide (D_2_O) was purchased from the Norell (Landisville, USA). Sodium 3-trimethlysilyl [2,2,3,3-d_4_] propionate (TSP) was obtained from the Cambridge Isotope Laboratories Inc. (Andover, MA). Phosphate buffer solution (PBS, 0.1 M, K_2_HPO_4_ / NaH_2_PO_4_ / NaCl /KCl, pH 7.2 - 7.6) was bought from the Boster Biological Technology Co. Ltd. (Wuhan, China). Ultrapure water was used for preparing all solutions. Rabbit monoclonal anti-caspase-3 antibody was purchased from Wuhan Servicebio Biological Technology Co. Ltd. (Wuhan, China). All other reagents were of analytical grade or higher.

### Animals and animal experiment design

Male Sprague-Dawley rats (8 weeks old, 220-240 g) were purchased from the Experimental Animal Center of the Chinese Military Medical Sciences Academy (License No. SCXK 2014-0013). The rats were housed under standard experimental conditions with room temperature at 23 ± 2° C, relative humidity 50 ± 10% and 12 h light-dark cycle. During the whole acclimatization and experimental period, rats had free access to food and tap water. All the experimental protocols used in the present study were approved by the Committee on the Ethics of Animal Experiments of Shanxi University (Approval No. SXULL 20180052).

To obtain the relationships of aging and constipation, we constructed two independent but related model groups. Rats were randomly divided into three groups (n=6), namely, the negative control (NC), the aging model group (AG) and the constipation model group (CG). Rats in the aging model group were subcutaneous injected (i.h.) with D-gal at a dose of 300 mg/kg/d for 11 consecutive weeks. Meanwhile, for the CG group, the model was started to be constructed at week 7 and lasted for 5 weeks. As such, all rats were raised for 11 weeks. Firstly, rats were intragastric injected (i.g.) with white vinegar of 5 mL at the first dose. Afterwards, the constipation rats were given with 3 mL/200g white vinegar and 5 mL/200g activated carbon iced water every two days. Rats in the NC group received i.h. saline and i.g. distilled water injection.

During the experiments, body weight gains were recorded once a week. Following a 11-week assimilation, fecal samples of individual rats were collected in metabolism cages between 09: 00 and 11: 00. All fecal samples were put into centrifuge tubes and storage at −80° C for the following analysis.

### Behavioral measurements

### Open-field test (OFT)

OFT was conducted to evaluate the autonomic inquiry-ability and tension of rats [52], which could indirectly reflect the spontaneous locomotor activity status of rats. Rats were placed in a bare box (150 × 150 cm^2^), which is equally divided into 5 × 5 cells. The OFT test lasted for five minutes, with the first minute of acclimatization followed by recording rat activity in four minutes. Hand-operated counters were used to score four major parameters, including the duration time in the center cell, the grooming time, the total distance and the rearing time. 75% ethanol was sprayed to eliminate possible bias caused by odors left by previous rats.

### Morris water maze (MWM) test

MWM test was performed to assess the abilities of spatial learning and memory of rats [53]. Tests were carried out at the end of the modeling period and last for five days. A round pool with 150 cm diameter × 70 cm high was used, which was filled with opacified water (24.0 ± 0.5° C). Rats were able to escape water by using a hidden platform which was below the surface of the water with 1 ~ 2 cm. All data within the maze were monitored using WMT-100S Morris water maze video analysis system (Tai League Software, Chengdu, China). In the four-day trials of positioning navigation experiment, rats were randomly put into a quadrant and allowed to freely find the platform. During the training period, rats were allowed to explore the platform for 2 times *per* day. The escape latency of each rat was recorded. If they failed to locate the platform within 60 sec, rats were led to the platform and allowed to stay on it for another 30 sec. The platform was removed in the spatial exploration experiment. The tracks of rats were observed within 60 sec. The spent time in the target quadrant and the number of platform crossings were recorded to evaluate the memory consolidation of rats.

### Defecation and excretion trials

The gastrointestinal transit trial was conducted to test the gastrointestinal motility of rats at week 0 and week 11 [53]. Specifically, after being fasted overnight for 16 h, all rats were given with a gavage of 1 mL self-configured ink containing 5% activated carbon powder and 10% Arabic gum as the indicator. Each rat was separately placed in a metabolic cage with compartments and allowed free access to food and water. For each rat, the time was recorded that from the beginning of providing rats with self-configured ink to the first defecation of black feces.

A pretest-posttest design was used to investigate the impacts of aging and constipation on urine volumes of rats. Each rat was separately placed in a metabolic cage and allowed free access to water. Urine samples were collected on ice to avoid being volatilized. After 12-hour metabolism, urine volume of each rat was measured and recorded.

### Gastric and small intestinal movement trials

Semi-solid nutritional paste was prepared and given to rats to simultaneously measure the gastric remnant rate and the gastrointestinal ink push rate. Each rat was given with a semi-solid nutrient paste containing PAC (1 mL/100g). After 30 min, all rats were humanely sacrificed. The whole stomach of each rat was immediately dissected. The gastric cardia and gastric pylorus were ligated. After the filter paper was dried, the total gastric weight (X) was obtained. The stomach of each rat was cut by a surgical scissor along the big curve of the gastric wall. Gastric contents were washed with distilled water. After removing water from the surface, the net gastric weight (Y) was weighed.

The emptying amount of semi-solid nutritional paste in stomach was calculated according to Formula A. Intestinal transit distances were determined by measuring the distance that the charcoal had migrated through the small intestine (from the pylorus to the cecum). The gastrointestinal ink push rates were calculated by Formula B [54].

$$\text{Gastric emptying rate}\;(\%)=\frac{\text{x}-\text{y}}{\text{z}}\times 100\%$$ A

Where X and Y was the total gastric weight and the net weight of stomach, respectively. Z was the weight of the semi-solid nutrient paste.

$$\begin{array}{l} \text{Gastrointestinal ink push rates (\%)}=\\ \frac{\text{The movement distance of ink}}{\text{Whole length of small intestine}}\times 100\% \end{array}$$ B

### Biochemical measurement

The indexes of oxidative stress of serum samples, including catalase (CAT), malondialdehyde (MDA), superoxide dismutase (SOD) and glutathione peroxidase (GSH-Px), were determined by using commercially available kits, according to the manufacturers’ instructions. Arbitrary enzymatic activity was expressed as the ratio of changes in optical absorbance to changes in protein content.

### Histological evaluation of hippocampus and the expression of caspase-3 in the hippocampus tissues of rats

To evaluate the histological changes in the hippocampus, the tissues were fixed in a 4% paraformaldehyde solution (pH = 7.4), and then were dehydrated and embedded in paraffin blocks. And then, samples were sectioned with 4.0 μm thickness. Rabbit monoclonal anti-caspase-3 antibody for the pro-form (GB11009-1, Servicebio, Wuhan, China) was utilized for immune-histochemical determination according to the manufacturer’s instructions. The immunohistochemical images were quantified by the number of positive cells in the CA3 region of hippocampal tissues by the Image J analysis software (Version 1.8.0). With the pixel area as the standard unit, we recorded the positive integrated optical density (IOD) of CA3 region and the corresponding tissue pixel area (TPA) in each slice. The areal density of positive cells was calculated by Formula C.

$$\text{Areal density}=\frac{\text{IOD}}{\text{TPA}}$$ C

### Metabolomics analysis

### Preparation of fecal samples

Fecal samples were extracted as described by Liu et al. with minor modifications [55]. Briefly, a total of 1000 μL of PBS buffer (0.1 M, pH = 7.4) contained 16.7% D_2_O (99.9% D) and 0.01% TSP was added to 100 mg thawed fecal samples. The mixture was then homogenized by vortex for 30 sec and dealt with ultrasonication cycles for 10 minutes. Ultrasound treatment was carried out in an ice bath in the form of ultrasonication (10 sec) – waiting (10 sec). Afterwards, vortex mixing was carried out again. The vortex samples were centrifuged at 4° C for 15 minutes at a speed of 13, 000 ×g. A 500 μL supernatant was diluted to 600 μL by adding D_2_O containing 0.01% TSP. The extract solutions of feces were transferred into 5 mm NMR tubes for analysis.

### ^1^H NMR spectra acquisition

^1^H NMR spectra were acquired at 298.2 K on a Bruker-AV 600 MHz NMR spectrometer (Bruker, Germany) equipped with a Bruker 5 mm PA BBO probe operated at 600.13 MHz ^1^H frequency. The fecal samples were analyzed using the Carr–Purcell–Meiboom–Gill (CPMG) NMR spectra. D_2_O was used for internal lock purposes. TSP (0.01%, w/v) was used as an internal standard. Each ^1^H NMR spectrum consisted of 64 scans requiring 5 min of acquisition time. The following parameters were set: spectral size of 65,536 points, spectral width of 12019.2 Hz, a relaxation delay (RD) of 1.0 s and pulse width (PW) of 14.

### ^1^H NMR spectral data preprocessing and multivariate data (MVD) analysis

^1^H NMR spectra were corrected for phase and baseline distortion and calibrated to TSP signal at 0.00 ppm using MestReNova software (Version 8.1.0, Santiago de Compostella, Spain). To reduce the complexity of data, spectra were segmented into bins with equal widths of 0.01 ppm each across the region of 0.60 – 9.20 ppm. The region of 4.68 – 5.17 ppm was discarded to eliminate the effects of water suppression.

To identify the aging-related and the constipation-related variations in the fecal metabolic phenotypes, NMR data were subjected to principal components analysis (PCA), partial least squares discriminant analysis (PLS-DA) and orthogonal partial least squares-discriminate analysis (OPLS-DA) by the Simca-P 13.0 software (Umetrics, Sweden).

PCA, as an unsupervised analysis manner, was adopted to observe the overall differences between the control group and the model groups. PLS-DA was then used to improve the discriminations between different groups. The models constructed were assessed by the parameters of model fitness (R^2^) and predictive ability (Q^2^) followed in a supervised manner with permutation tests (No. 200 cycles). The values of these parameters closing to 1.0 indicate a reasonably good differentiating, fitness and prediction of the constructed models. OPLS-DA, a supervised extension of PCA, was used to screen variables that were responsible for group separations. The corresponding S-plot was used to assess and identify the contributions of metabolites to the separations of metabolic profiles. The models were validated using CV-ANOVA tests with a significant level of *P* < 0.05.

### Identification of differential metabolites and construction of metabolic pathways

Resonance assignments were performed by comparison with previous data [2, 55, 56] and online databases, including the Human Metabolome Data Base (HMDB, http://www.hmdb.ca), the Biological Magnetic Resonance Data Bank (BMRB, http://www.bmrb.wisc.edu) and our in-house databases. Metabolites were firstly highlighted by the S-plot and the values of variable importance (VIP) for projections. In the S-plot, the further the distance away from the center, the greater the metabolites contribute to the separation. Those metabolites with VIP > 1.2 and the presence of a statistically significant difference (*P* < 0.05) were regarded as potential biomarkers.

In addition, a SUS-plot was performed which combines two predictive loadings obtained from two different OPLS-DA models, both sharing a common control group. Each variable has one coordinate from each model. Specifically, variations along the abscissa axis and the ordinate axis of the SUS-plot are involved in aging and constipation, respectively. This approach is of significance in terms of showing in particular the discrimination between aging-related metabolites and constipation-related metabolites.

To confirm the targeted metabolic pathways and to visualize metabolic networks, metabolic pathways were constructed and analyzed by the Metabolomic Pathway Analysis (MetPA) based on the MetaboAnalyst 4.0 platform (http://www.metaboanalyst.ca) and the KEGG pathway database (http://www.kegg.jp/kegg/pathway.hml).

### Correlation analysis

The Pearson’s correlation analysis was further employed to assess the associations among potential biomarkers related to aging, as well as to constipation. The correlation coefficients (r) and the *P* values, depicting the degrees of correlations between aging-related and constipation-related behaviors or metabolites, were used to determine contribution degrees of constituents to the diseases. Correlation coefficients were ranged from 1.0 (maximum positive correlation) to −1.0 (maximum anti-correlation), with a value of 0 representing no correlation. In the cross-correlation heat-map, red indicated positive correlations while blue represented negative correlations.

### Gene ontology (GO) enrichment analysis and pathway enrichment analysis

Aging-related human genes and constipation-related human genes were collected from the Gencards Database (https://www.genecards.org/). The screening of disease targets was scored where the screening score value of aging and constipation was set as 40 and 10, respectively. GO analysis and pathway enrichment analysis were conducted by the functional annotation tool of DAVID Bioinformatics Resources 6.7 (http://david.abcc.ncifcrf.gov/) to predict metabolic pathways that related to aging and constipation, respectively (*P* < 0.05). Terms with thresholds of Count 2 and Expression Analysis Systematic Explorer (EASE) scores 0.1 were chosen in clustering functional annotation. The target-pathway network of a disease was established by Cytoscape 3.7.2 software.

### Statistical analysis

All experiments were repeated for at least three times. Data of behavioral parameters were expressed as mean ± standard error (S.E.). One-way analysis of variance (ANOVA) was used to analyze significant differences of behavior data and relative metabolite concentrations among groups (SPSS statistics 25.0, IBM, Armonk, NY, USA). The broken line graphs, column bar graphs, and box-plots were drawn by the GraphPad Prism software (Version 7.0, San Diego, CA, USA). A value of *P* < 0.05 was considered to be statistically significant.
